# Dynamic Amplification of Jack-Up Platforms Subjected to Non-Gaussian Wave Loads

**DOI:** 10.6028/jres.099.043

**Published:** 1994

**Authors:** Jørgen Junchcr Jensen

**Affiliations:** Department of Ocean Engineering, Technical University of Denmark, Building 101 E, DK-2800 Lyngby, Denmark

**Keywords:** diffusion theory, dynamic amplification, Jack-up platforms, non-Gaussian waveloads, nonlinear wave response

## Abstract

Jack-up platforms are sensitive to dynamic amplification in waves because their fundamental period can be as high as 7 s–8 s. Where as the dynamic motion of the platforms is rather well described by linear theory the excitation depends nonlinearly on the wave height. The stochastic wave loading is thus far from being normally distributed.

In this paper dynamic amplifications obtained by the diffusion theory extended to cover nonnormal excitations are compared with available time simulation results in irregular seaways. Generally, the time simulations seem to yield responses less nonlinear in the wave heights than the responses estimated from the diffusion theory. Explanations for these discrepancies are discussed and mainly attributed to a proper choice of total damping.

## 1. Introduction

**Fig. 1:**
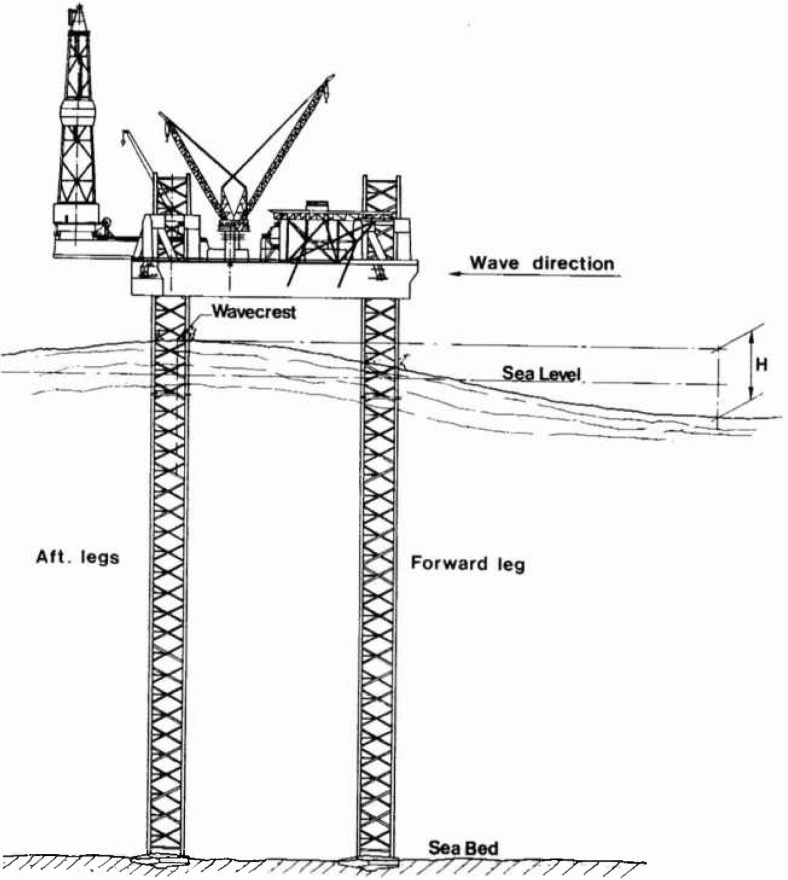
Jack-up platform with cantilever.

The jack-up drilling rig concept, Fig. 1, has proved to be very convenient in the exploration for oil and gas in offshore areas. Therefore, requests are made for designs able to operate in increasing water depths. Due to their sizes and independent leg configuration the natural periods of their lowest vibration modes become comparable with the dominant wave periods in the design sea states. As an example jack-up rigs with leg lengths of 160 m, hull masses of the order 15,000 t and lowest natural periods around 8 s are currently under construction. For such structures dynamic amplification of the wave load responses is certainly to be expected.

The wave loading on the legs can be estimated using Morison’s equation. Usually, the legs are trusslike, with each leg consisting of three (or four) vertical chords connected by horizontal and oblique bracing members. The diameters of the individual members are so small that the wave loads on the legs become drag dominated. Alternative designs for smaller platforms have considered circular cylindrical legs, yielding inertia-dominant waveloads [1]. For larger platforms the circular cylindrical leg design is not feasible as the loadings and thereby the required amount of steel are so much higher than for the truss leg design that it cannot be counterbalanced by lower production costs.

A drag-dominated wave load implies a loading which is nonlinear in the wave height. Furthermore, the integration of the wave load up to the actual position of the wave elevation on a leg and the non-symmetry (Stoke’s 5th order wave) of the wave profile magnify these nonlinearities in the base shear, the overturning moment and associated responses.

The structural stiffness of the jack-up rig in the lowest vibration mode is characterized by the leg stiffness, the distance between the legs, the bottom support conditions, the distance *L* from sea bottom to the platform deck and the leg-jack-house flexibility. The global vibration pattern is normally beamlike with a maximum horizontal deck deflection of the order of 1 *%–*2 *%* of *L* in the design sea states. A linear structural analysis would therefore normally suffice. However, the additional overturning moment in the deflected state due to the high axial leg loads from the deck mass must be included by the so-called *P – δ* effect and, in a dynamic analysis, by reducing the leg bending stiffness.

A jack-up rig is a highly stressed structure. Therefore, it is important that an accurate structural evaluation is performed. Such an analysis should be done, not only when designing a new structure, but also when a jack-up rig is moved to a new location. In order to get uniform and reliable site approval procedures, a large study was initiated by a group of companies involved in jack-up design and operations, A summary of this project is presented in [2]. Generally three different methods are applied:

- Single degree of freedom methods (SDOF).
- Frequency domain methods.
- Time-domain methods.

In the first procedure the quasistatic solution, determined by neglecting the motion of the platform, is amplified by a dynamic amplification factor (DAF) calculated by the classical SDOF formula. This will introduce errors due to the nonlinearities in the wave loading and several approximative procedures have been used [3]–[5], aiming at reducing these errors.

The frequency domain methods rely on a suitable linearization of the wave loads with the wave height. The resulting linear dynamic system is then solved exactly. The non-Gaussian behavior of the extreme values is then simply estimated by multiplying the standard deviation of the linear response with a factor depending on the ratio between the root-meansquare values of the drag and inertia terms in the wave loads.

Due to the assumptions inherent into above-mentioned methods, time simulation procedures are often used. For a specific stationary stochastic sea state random time signals of wave elevation and corresponding wave kinematics are generated, typically by superposition of first order (Airy) wave components. A structural analysis of the jackup-rig including dynamic and nonlinear effects is then carried out using time steps of the order of 0.5 s. The main drawback in this method is that due to excessive computational costs only a limited number of time simulations, each covering a few hours, can be generated. The extrapolation of these results to extreme value predictions for design approval can be difficult. Several applications of time simulation procedures to jack-up rigs have been published [1]–[2], [4]–[7].

In a previous paper by the author [8], an alternative method has been developed. The method is based on exact solution of a linear single-degree-of-freedom system subjected to a non-Gaussian excitation. Like in the SDOF method the present method also needs the nonlinear quasistatic response as input but now the dynamic effects are calculated much more consistently. The present procedure yields all required statistical moments of the response, which makes extreme value predictions very easy. This is done without using any stochastic linearization procedures as required in the frequency domain method. Finally, compared to time simulation procedures, the present method is much faster to apply and does not have the problems with extreme value predictions inherent in time simulation procedures.

The aim of the present paper is to evaluate the proposed procedure [8] by comparing results with those obtained from time simulation procedures. Previous comparisons [8], [9], with results based on a usual SDOF procedure have been very favorable as well as have been comparisons with time simulation results for a large offshore jacket structure [10]. In the next section the present stochastic dynamic procedure is described. Then it is applied to data presented in [5], obtained using a time simulation procedure and the importance of the various approximations and different modelings is discussed.

## 2. Stochastic Dynamic Analysis

For a linear single-degree-of-freedom system the equation of motion for the response *Y*(*t*) can be written

$$\ddot{Y}(t)+2\zeta_{0}\omega_{0}\dot{Y}(t)+\omega_{0}^{2}(t)=\omega_{0}^{2}Y_{0}(t),$$

where *ω*_0_ and *ζ*_0_ is the natural frequency and damping ratio, respectively. Time is denoted by *t* and differentiation with respect to *t* by (·). The function *Y*_0_(*t*) is seen to be the quasistatic response obtained neglecting the dynamic behavior of the structure (ω_0_→∞).

If *Y*_0_(*t*) represents a global jack-up response variable like the base shear or overturning moment, then it has been shown, [3], [8]–[10] that it can quite accurately be represented by a polynomial description in terms of the wave height *H.*

$$Y_{0}(t)=\sum_{i=0}^{n}A_{i}h{\left(t\right)}^{i}$$ (2)

$$h(t)=\frac{H}{2}\cos\left[2\pi \frac{t}{T}+\epsilon \right],$$ (3)

when the jack-up is subjected to a regular long-crested wave. The coefficients *A_i_* will depend on the platform geometry, the water depth, the wave theory applied and the current profile. No closed-form solution exists and the actual values of *A_i_* must be derived by curve fitting from the numerical results.

The wave period *T* in the applied regular wave and in equivalent wave elevation *h* is taken to be uniquely given by the wave height *H*, using for instance Odland’s formula [11]

$$T=1+4.1H^{0.4},$$ (4)

with *T* in seconds and *H* in meters.

In a stationary stochastic sea state with significant wave heights *H*_s_ the individual wave heights *H* have been found to be Rayleigh distributed with a root-mean-square value close to $H_{s}/2\sqrt{2}$. Furthermore, the phase lag *ϵ* in Eq. (3) can be taken to be uniformly distributed. Then the parameter *h*, Eq. (3), becomes normal distributed with zero mean and standard deviation equal to *H*_s_/4. Thereby, the stochastic equivalent of Eq. (2) becomes

$$Y_{0}(t)=\sum_{i=0}^{n}a_{i}U{\left(t\right)}^{i}$$ (5)

with

$$a_{i}=A_{i}{\left(H_{s}/4\right)}^{i}$$ (6)

and where *U*(*t*) is a standard Gaussian process with zero mean and unit variance. In most cases a cubic polynomial, *n* = 3, will suffice.

Clearly the curve fitting and the specific values of *T = T*(*H*) used to obtain the quasistatic response description, Eq. (2), impose some inaccuracies in the coefficient *a_i_.* Therefore, if time simulation results are available for the stochastic quasistatic response, *Y*_0_, then these results could be used directly to generate proper values of *a_i_.* For example, from the four lowest statistical moments: mean, standard deviation, skewness, and kurtosis, it is straightforward to determine the four coefficients *a_i_* in a cubic description of *Y*_0_(*t*) [8]. This possibility will be considered in the next section.

The solution of Eq. (1) with the right hand side given by Eq. (5) will be based on the theory of diffusion processes. Therefore the forcing function *ξ*(*t*) must be a normal white-noise process with a covariance function satisfying

E[ξ(t)ξ(t+τ)]=2πSδ(τ), (7)

where *S* is the spectral density of *ξ* and *δ*(*τ*) is Dirac’s delta function. A constant spectral density is a very poor approximation for a wave load process *U*(*t*) and the standard procedure to overcome this problem is to pass the white noise process *ξ*(*t*) through a filter defined by

$$\ddot{\eta }+2\zeta_{g}\;\omega_{g}\;\dot{\eta }+\omega_{g}^{2}\eta =\xi .$$ (8)

Thereby, the spectral shape *S_η_* of *η* becomes

$$S_{\eta }(\omega )=\frac{2S}{{\left(\omega^{2}-\omega_{g}^{2}\right)}^{2}+{\left(2\zeta_{g}\;\omega_{g}\;\omega \right)}^{2}};\;\omega \geq 0.$$ (9)

**Fig. 2:**
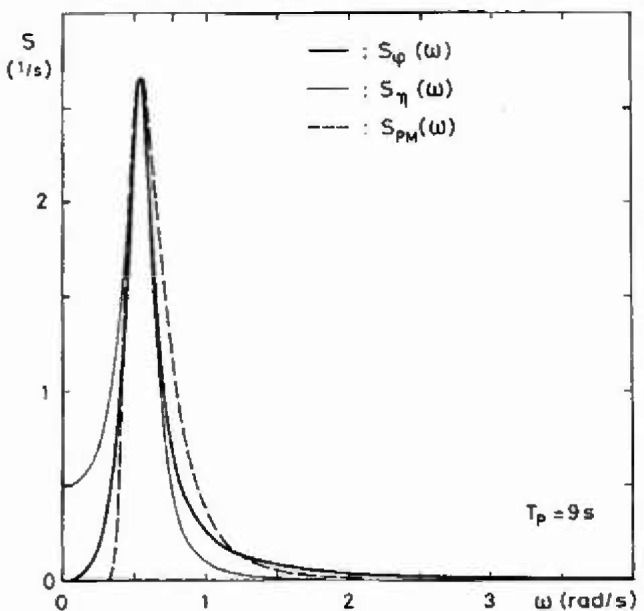
Comparison between the normalized spectra *S_η_*(*ω*). *S*_φ_(*ω*) and *S*_PM_(*ω*).

Compared to the usual wave spectra of the Pierson-Moskowitz type the spectrum *S_η_*(*ω*) has the disadvantage that *S_η_*(0)≠0. The spectral shape *S*_φ_(*ω*)

$$S_{\varphi }\;(\omega )=S_{\eta }\;(\omega )\;{\left(\omega /\omega_{g}\right)}^{2}$$ (10)

of the process

$$\varphi (t)=\dot{\eta }\;(t)/\omega_{g}$$ (11)

much better resembles the Pierson-Moskowitz spectrum. This is illustrated in Fig. 2, where the normalized spectra *S_η_*(*ω*), *S_φ_*(*ω*) are compared with the normalized Pierson-Moskowitz spectrum

$$S_{\text{PM}}(\omega )=4\tau_{p}^{-4}\;\omega^{-5}\;\exp\left(-{\left(\tau_{p}\omega \right)}^{-4}\right)\;;\;\omega \geq 0.$$ (12)

Here

$$\tau_{P}=\frac{T_{P}}{2\pi }{\left(\frac{4}{5}\right)}^{1/4},$$ (13)

where *T*_p_ is the spectral peak period for the sea state. The normalizations are such that all three spectra have a unit variance implying that

$$S=\frac{2\zeta_{g}\;\omega_{g}^{3}}{\pi }$$ (14)

for both *S_η_ and S_φ_.*

Furthermore, the spectral parameters *ω*_g_ and *ζ*_g_ are chosen such that the peak values and peak frequencies for all three spectra coincide [8], yielding

$$\begin{array}{c} S_{\eta }(\omega ):\;\omega_{g}=1.052\frac{2\pi }{T_{P}};\;\zeta_{g}=0.221\\ S\varphi (\omega ):\;\omega_{g}=\frac{2\pi }{T_{P}};\;\zeta_{g}=0.222 \end{array}$$ (15)

In the following normal process *U*(*t*) in Eq. (5) will be taken as

U(t)=φ(t) (16)

Eqs. (1), (5), (8), and (11) can be written as Ito differential equations

$$\dot{Z}=C(Z(t))+W(t),$$ (17)

where

$$Z={\left\{Y,\dot{Y}/\omega_{0},\eta ,\varphi \right\}}^{T}\equiv {\left\{Z_{1},Z_{2},Z_{3},Z_{4}\right\}}^{T}$$ (18)

and

$$C(Z)=\left[\begin{array}{l} \omega_{0}Z_{2}\\ -2\;\zeta_{0}\;\omega_{0}\;Z_{2}-\omega_{0}Z_{1}+\omega_{0}\sum_{i=0}^{n}a_{i}Z_{4}^{i}\\ \omega_{g}Z_{4}\\ -\;2\;\zeta_{g}\;\omega_{g}\;Z_{4}-\omega_{g}\;Z_{3} \end{array}\right]$$ (19)

$$W(t)={\left\{0,0,0,\;\xi (t)/\omega_{g}\right\}}^{T}.$$ (20)

Since *W*(*t*) satisfies the white noise property

E[W(t)W(t+τ)]=Dδ(t), (21)

it follows from diffusion theory, e.g., [12] that the statistical mean value *E*[*g*(*Z*)] of any function *g* of *Z* satisfies

$$\begin{array}{c} \sum_{i}E\left[C_{i}(Z)\frac{\partial g}{\partial Z_{i}}\right]+\\ \frac{1}{2}\;\sum_{i}\sum_{j}D_{ij}E\left[\frac{\partial^{2}g}{\partial Z_{i}\;\partial Z_{j}}\right]=0 \end{array}$$ (22)

in a stationary sea state. In the present case *i*, *j* = 1, 2, 3, 4 and only the *D*_44_ component in the 4×4 matrix *D* is different from zero.

As the vector *C*(*Z*) is given in polynomial form, it is straightforward to apply the procedure given by Krenk and Gluver [12] to obtain exact values for the statistical moment of *Y.* Here only the four lowest moments are determined and used to define uniquely a cubic polynomial approximation for *Y*(*t*), [8]

$$Y(t)=C_{0}+C_{1}\;U(t)+c_{2}\;U{\left(t\right)}^{2}+c_{3}\;U{\left(t\right)}^{3}.$$ (23)

Extreme values of *Y*(*t*) are finally obtained by replacing *U*(*t*) with corresponding extreme values, that is by $\sqrt{2\;\ln\;N}$ for the most probable largest peak among *N* peaks.

## 3. Numerical Results

For a linear single-degree-of-freedom system subjected to a Gaussian excitation, a dynamic amplification factor can be defined as [11]

$$\begin{array}{r} \text{dynamic}\;\text{amplification}\;\text{factor}=\\ \frac{{\left[\int_{0}^{\infty }\;\psi^{2}(\omega )\;S(\omega )\;d\omega \right]}^{1/2}}{\sigma_{g}}, \end{array}$$ (24)

where *ψ* is the classical dynamic amplification factor

$$\psi (\omega )=\frac{\omega_{0}^{2}}{\sqrt{{\left(\omega_{0}^{2}-\omega^{2}\right)}^{2}+{\left(2\zeta_{0}\;\omega \;\omega_{0}\right)}^{2}}}$$ (25)

and where *S*(*ω*) is the spectral density of the quasistatic response (the excitation) *Y*_0._ Furthermore, *σ*_3_ is the standard deviation of the excitation given by

$$\sigma_{s}^{2}=\int_{0}^{\infty }S(\omega )\;d\omega .$$ (26)

**Fig. 3:**
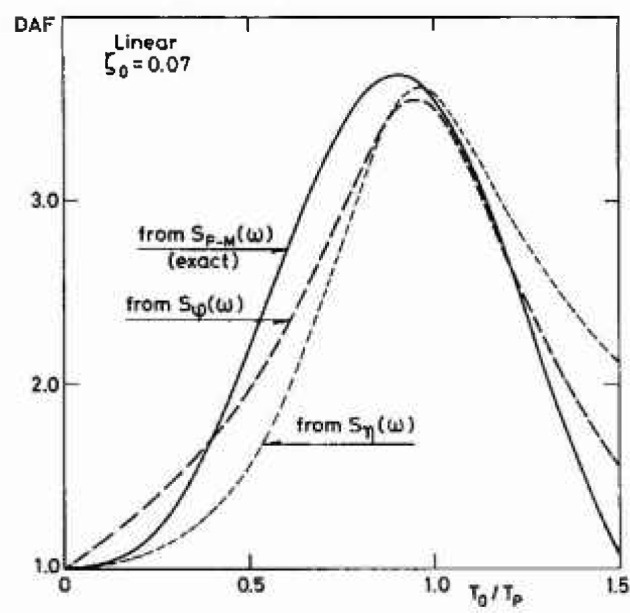
Dynamic amplification factor (DAF), Eq. (24), for the standard deviation of a linear one degree-of-freedom system, using different excitation spectral densities.

Examples of dynamic amplification factors determined by Eq. (24) are shown in Fig. 3. Three spectral densities *S*(*ω*) are used, the Pierson-Moskowitz spectrum, Eq. (12), the spectrum *S_η_*(*ω*), Eq. (9), and the spectrum *S_φ_*(*ω*), Eq. (10). The damping ratio is taken to be *ζ*_0_ = 0.07 and it is seen that at resonance the dynamic amplification factor is only about half the value of *ψ*(*ω*_0_) = 1/2 *ζ*_0_.

For a linear system subjected to a Gaussian excitation the same dynamic amplification factor will apply to both standard deviations and extreme values. Typical values of the fundamental period *T*_0_=2*π*/*ω*_0_ are around 8 s for large jack-up rigs whereas the peak spectral period *T*_p_ in the design sea state is about 16 s. From Fig. 3 one could then expect dynamic amplification factors in the vicinity of 2. However, most time simulation results [4]–[7], yield dynamic amplification factors for the extreme values much lower and even sometimes below 1. In the following this difference will be discussed using data for the example jack-up rig considered in [5].

**Fig. 4:**
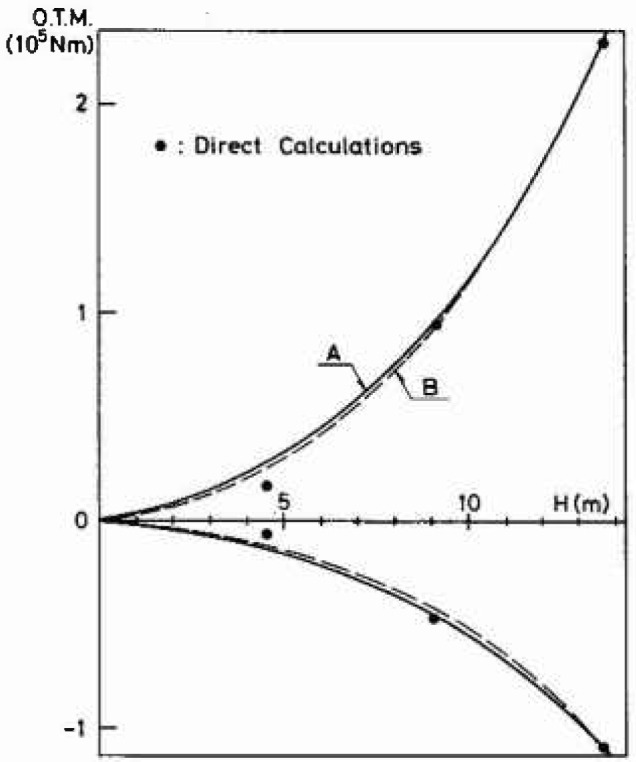
Overturning moment for the jack-up-rig considered [5], subjected to a regular long-crested stretched Airy wave.

First deterministic, quasistatic results for this jack-up rig were computed using both the Stretched Airy and the Stoke’s 5th order wave theory. The results obtained for the overturning moment (OTM) using the Stretched Airy wave theory are shown in Fig. 4. Corrections for *P-δ* effects have been made. The wave period *T* is taken in accordance with Eq. (4). The sensitivity of the calculated results to the choice of *T* is exemplified in Table 1. It is seen that minor variations in *T* around the value given by Eq. (4) do not change the overturning moment significantly.

Two different curve fitting procedures have been used in Fig. 4 to generate cubic polynomial representations (A, B) of the overturning moment as function of the deterministic wave height *H.* Similar curves are obtained for the base shear and also when using the Stoke’s 5th order wave theory. All these results are expressed in terms of coefficients *A_i_* to be used in Eq. (2).

The coefficients *A_i_* are then used in Eq. (6) to obtain values of *a_i_* valid for the stationary stochastic design sea state. From these coefficients the four lowest statistical moments are calculated by the procedure given in [8]. The results are given in Tables 2 and 3 and compared with those presented in [5] from a quasistatic time simulation procedure. Furthermore, these tables contain the dynamic results determined by the present stochastic dynamic procedure and by dynamic time simulations in random seaways [5]. From Table 2 it is clear that the choice of wave theory and curve fitting procedure has only a marginal influence on the quasistatic overturning moment. Therefore Table 3 for the base shear only contains resutts for one of these choices. Also it appears that the statistical moments calculated from the deterministic results using Eqs. (2)–(6) are remarkably close to those found from time simulations except perhaps for the kurtosis which is somewhat lower in the time simulations.

The only additional information needed to calculate the dynamic responses by the present stochastic dynamic procedure is the fundamental period *T*_0_ and the total damping ratio *ζ*_0_, see Eq. (1). For the example jack-up rig *T*_0_=8.45 s whereas the damping ratio is specified to 0.05 [5]. However, the time simulations are carried out in [5] using a single-degree-of-freedom formulation which includes coupled fluid-leg interaction terms. These terms will reduce the dynamic response and therefore act as additional (hydrodynamic) damping. The total damping in these time simulations must thus be greater than 0.05. In the present calculations, Eq. (1), (7)–(23), the damping ratio has been taken to be *ζ*_0_=0.05. The consequences of larger actual damping will be discussed later.

It is seen from Tables 2 and 3 that the dynamic amplification factor is nearly the same whether the quasistatic input is taken from Eqs. (2)–(6) or from the quasi static stochastic time simulations performed in [5] (the results marked by (*)). The difference in kurtosis is apparently not important.

For both the base shear and the overturning moment the dynamic amplification factors for the standard deviation turn out to be around 2.4 whereas the dynamic amplification factor for the most probable largest response peak among *N* = 1000 peaks becomes 1.36. As *T*_0_/*T*_p_ = 8.45/15.5 = 0.54 the dynamic amplification factor for the standard deviation is seen to be in accordance with Fig. 3 taking into account that the damping in the present example is *ζ*_0_=0.05. Tables 2 and 3 also show that the dynamic effects tend to reduce the skewness and kurtosis making the response more Gaussian than the quasistatic response. Thereby, the dynamic amplification factors for the extreme values become smaller than for the standard deviation with decreasing values for increasing values of *N.*

The most severe disagreement between the results from the present stochastic dynamic procedure and the time simulations is clearly in the dynamic amplification factors. They are consistently smaller in the time simulations.

Before looking after possible explanations it should be stressed that the above results only concern one specific jack-up rig. Other results obtained by time simulations have shown larger dynamic amplification factors. For instance the dynamic amplification factor for standard deviation of the overturning moment is found in Ref. [6], Figs. 7 and 11 to be 5.583/2.521 =2.21 for a comparable jack-up rig and sea state. Also [4] shows dynamic amplification factors around two without specifying precisely the extreme value level.

One source of uncertainty is the damping ratio. In the present stochastic dynamic procedure the total damping has to be specified whereas in the time simulation procedure the hydrodynamic damping is automatically taken into account by the relative velocity terms in Morison’s equation. Therefore, it could be interesting to see how much the total damping should be increased before results in accordance with the time simulations are obtained. In Table 4 such results are shown and it is seen that first for a total damping ratio of about 20 *%* good agreement on dynamic amplification factors is obtained. Such damping is rarely expected in real jack-ups although [4] presents experimental values around 10 % for a model test. A total damping as low as 2.2 *%* has on the other hand been estimated from full scale measurements [14]. As mentioned previously, the stochastic dynamic time simulation procedure in [5] includes 5 *%* damping in addition to some damping from fluid-structure interactions (Eq. (26) in [5]). This must result in an effective total damping in the time simulation results greater than 5 % but how much greater it is not possible to say.

A further verification of the present procedure will clearly require more detailed comparisons with time simulation results including estimations of the total damping in the time simulations as function of the severity of the sea state. Until then it seems reasonable to assume that if the total damping is known then the present procedure will yield results with uncertainties mainly related to the assumption of a single degree-of-freedom system. Note that *P* – *δ* effects are included in *T*_0_ through a reduced leg stiffness [11].

**Fig. 5:**
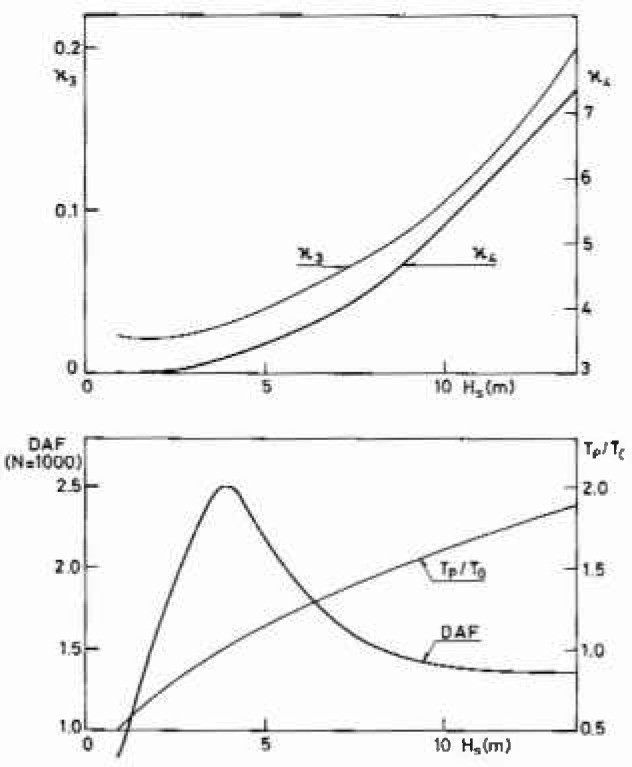
Skewness *κ*_3_, kurtosis *κ*_4_ and dynamic amplification factors for the dynamic overturning moment as function of the significant wave height.

**Fig. 6:**
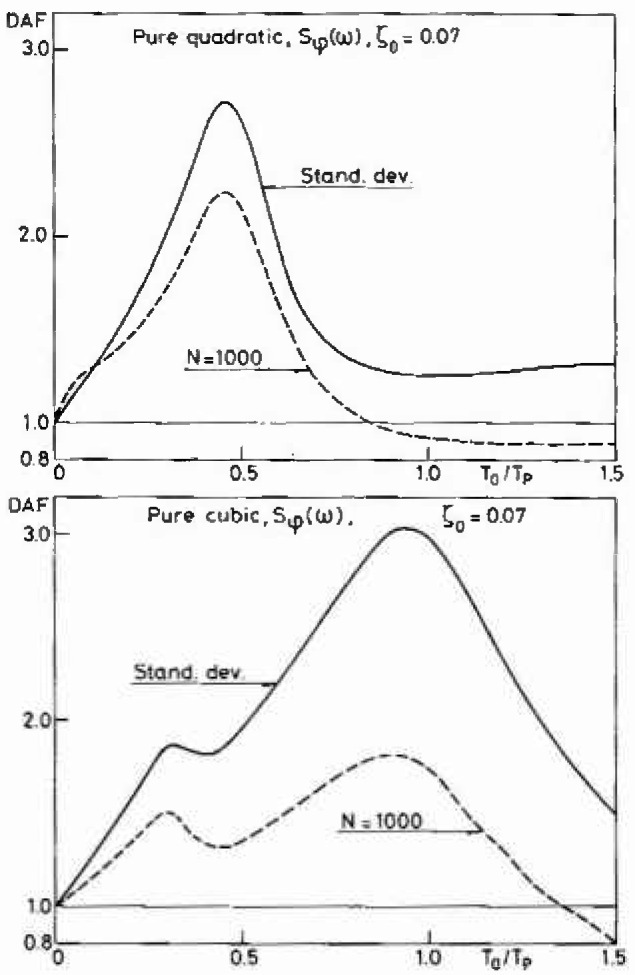
The dynamic amplification of the standard deviation and the most probable largest peak among 1000 peaks for a pure quadratic and a pure cubic excitation.

To illustrate the potential of the present procedure, Fig. 5 shows the variation with sea state of the non-Gaussian behavior and of the dynamic amplification factor for the most probable peak value among 1000 peaks for the overturning moment. In particular, one should note that even in extreme sea states some dynamic amplification occurs. There are two reasons for this. First the stochastic sea state averages out the classical dynamic amplification factor as shown in Fig. 3. Secondly, the non-Gaussian parts of the quasistatic excitation *Y*_0_ are amplified differently. In Fig. 6 the dynamic amplifications associated with a pure quadratic and a pure cubic excitation are shown. The linear excitation, Fig. 3, has a maximum dynamic amplification factor for *T*_0_/*T_p_* ≃1, whereas the quadratic excitation yields maxima for *T*_0_/*T*_p_ ≃0.5 and the cubic excitation maxima for *T*_0_/*T*_p_ ≃0.33 and *T*_0_/*T*_p_ ≃1. Thus depending on the relative magnitude of the linear (*a*_1_), quadratic (*a*_2_) and cubic (*a*_3_) terms in the excitation *Y*_0_, Eq. (5), the largest dynamic amplification factor can appear within a range of *T*_0_/*T*_p_ values. For the example considered here in Fig. 5, the linear and especially the cubic terms dominate yielding a maximum dynamic amplification factor when the spectral peak period *T*_p_ gets close to the fundamental natural period *T*_0_. Note, however, that most jack-up rigs have *T*_0_/*T*_p_≃0.5 in the design sea state which is where the dynamic amplification factor from the quadratic term is largest. This is the reason why the dynamic amplification factor in Fig. 5 levels off for *H*_3_ around 13.7 m (45 ft).

**Fig. 7:**
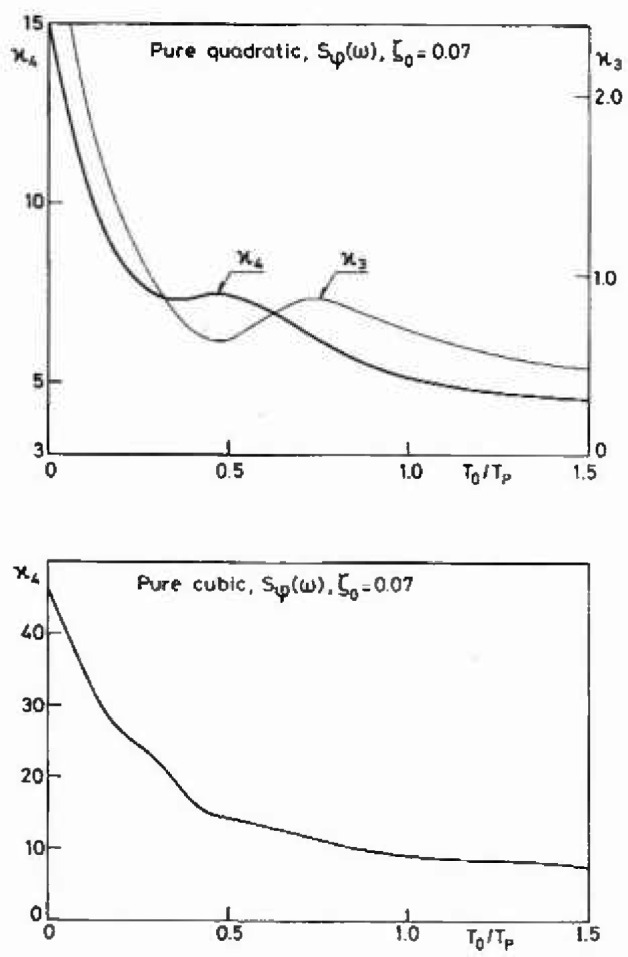
Skewness *κ*_3_ and kurtosis *κ*_4_ for a dynamic system subjected to a pure quadratic and a pure cubic (*κ*_3_ = 0) excitation.

Finally, Fig. 7 shows for sake of completeness the variations of the skewness and the kurtosis for a dynamic system subjected to a pure quadratic and a pure cubic excitation. Note that a quadratic excitation has $\kappa_{3}=2\;\sqrt{2}$, *κ*_4_ = 15 whereas the cubic excitation has *κ*_3_=0, *κ*_4_ = 46.2. The change towards a Gaussian behavior is clearly significant already for *T*_0_>0.1 *T*_p_.

## 4. Conclusions

A procedure able to predict dynamic global responses of jack-up rigs subjected to wave loads in stationary stochastic seaways has been described.

The procedure consists of three steps:

- determine the wave load response using a suitable nonlinear regular wave theory neglecting the motion of the platform,
- fit a polynomial in the wave height through the calculated response maxima and minima,
- assume a deflection mode in the form of the first horizontal vibration mode and solve the corresponding equation of motion in stationary sea states using the theory of diffusion processes.

For an example jack-up rig it is observed that the dynamic amplification quite significantly changes the statistical behavior of the response toward a Gaussian process. Also, a significant dynamic amplification is found in the extreme sea states where the spectral peak period is about twice the lowest natural period. These results are in agreement with previous findings [1]–[13] using various formulations and jack-up geometries.

Comparisons with time simulations performed for the example jack-up rig considered in [5] have indicated that the main uncertainty in the present procedure relates to a proper choice of total damping. In order to clarify this point estimates of total damping ratios from time simulation results would be extremely helpful. Such dampings would include fluid-structure interaction effects and will vary with time. Suitable average values must then be defined. In this context also doubts expressed on the use of the relative velocity term in Morisons equation should be mentioned [13].

## Figures and Tables

**Table 1:** Sensitivity of overturning moment (OTM) to wave period *T, H* = 13.7 m (45 ft), Stoke’s 5th order wave

Wave period *T* (s)	max OTM (10^5^ Nm)	min OTM (10^5^ Nm)
9.854	3.108	−0.811
11.261	2.635	−0.941
12.690 [Eq. (4)]	2.428	−1.112
14.077	2.457	−1.261

**Table 2:** Statistical moments and dynamic amplification factors (DAF) for the overturning moment in the design sea state, *H*_s_=12.8 m, *T*_p_=15.5 s

Overturning moment	Stoke’s 5th		Stretched Airy		Time simulation	Based on (*)

	fit A	fît B	fit A	fit B		
**Quasistatik** Mean/stand. dev.	0.197	0.198	0.192	0.193	0.200 (*)	
Skewness	2.60	2.77	2.50	2.61	2.99 (*)	
Kurtosis	25.7	29.7	24.3	27.0	18.4 (*)	

**Dynamic** Mean/stand. dev.	0.081	0.082	0.079	0.080	0.128	0.080
Skewness	0.17	0.19	0.16	0.18	1.29	0.19
Kurtosis	6.89	7.60	6.67	7.15	8.40	5.30
DAF/stand. dev.	2.43	2.41	2.43	2.41	1.49	2.50
DAF (*N* = 1000)	1.36	1.36	1.36	1.36	1.08	1.39

**Table 3:** Statistical moments and dynamic amplification factors (DAF) for the base shear in the design sea slate, *H*_1_= 12.8 m, *T*_p_=15.5 s

Base shear (BS)	Stoke’s 5th	Time simulation [5]	Based on (*)
**Quasistatic**			
Mean/stand. dev.	0.163	0.160 (*)	
Skewness	2.43	2.23 (*)	
Kurtosis	32.2	13.7 (*)	

**Dynamic**			
Mean/stand. dev.	0.068	0.122	0.065
Skewness	0.17	1.34	0.12
Kurtosis	8.18	9.01	4.71
DAF (stand. dev.)	2.40	1.24	2.46
DAF (*N* = 1000)	1.37	1.05	1.83

**Table 4:** Statistical moments and dynamic amplification factors (DAFs) for the overturning moment in the design sea state, (*H*_1_=12.8 m, *T*_p_= 15.5 s) as function of total damping ξ_0_

*ξ*_0_	Mean *μ*	Stand. dev. *σ*	Skewness *κ*_3_	Kurtosis *κ*_4_	DAF	
					Stand dev.	*N* = 903
0.05	20.5	258	0.19	5.3	2.50	1.39
0.10	20.5	193	0.52	7.1	1.87	1.26
0.15	20.5	165	0.90	8.4	1.60	1.19
0.20	20.5	148	1.25	9.4	1.44	1.13

Qunsistalic time sim. [5]	20.5	103	2.99	18.4	1.00	1.00

Dynamic time sim. [5]	19.6	153	1.29	8.4	1.49	1.08
